# Assessment of Physical Disability After Three Months in Patients Recovered From COVID-19: A Cross-Sectional Study

**DOI:** 10.7759/cureus.21618

**Published:** 2022-01-25

**Authors:** Ravi Gaur, Satyasheel Asthana, Rajkumar Yadav, Rambeer Ghuleliya, Deepak Kumar, Minhaj Akhtar, Nitesh Gonnade, Arun Choudhary, Merrin M Mathew, Neeru Gaur

**Affiliations:** 1 Physical Medicine and Rehabilitation, All India Institute of Medical Sciences, Jodhpur, IND; 2 Physical Medicine and Rehabilitation, Dr. Ram Manohar Lohia Institute of Medical Sciences, Lucknow, IND; 3 Physical Medicine and Rehabilitation, All India Institute of Medical Sciences, Rishikesh, IND; 4 Medicine, All India Institute of Medical Sciences, Jodhpur, IND; 5 Anaesthesiology, Fortis Escorts Hospital, Jaipur, IND

**Keywords:** dyspnoea, long covid, post covid fatigue, post covid disability, disability, whodas 2.0, covid-19 infection

## Abstract

Purpose: This study was done to assess the extent of disability in coronavirus disease 2019 (COVID-19) survivors using the World Health Organization Disability Assessment Schedule 2.0 (WHODAS 2.0).

Material and methods: This was a cross-sectional study with convenient sampling. Institutional ethical clearance was taken. Informed consent was taken from all patients. Disability assessment was done using WHODAS 2.0. All patients were initial reverse transcriptase-polymerase chain reaction (RT-PCR) positive for COVID-19. Patients with neuromuscular deficits or who were taking medication for psychiatric illness before getting infected with COVID-19 were excluded from the study.

Results: Fatigue followed by dyspnea was the most common reported symptom after three months of COVID-19 infection. COVID-19 survivors with fatigue or dyspnea had a more significant disability as compared to other patients. Females had a more significant disability when compared to males. We did not find any significant disability in COVID-19 survivors after three months of disease based on body mass index, hospitalization, diabetes, and oxygen requirements.

Conclusion: COVID-19 survivors suffered from significant disability after three months of disease especially females and survivors with fatigue or dyspnea. Recognizing post-COVID-19 sequelae and the availability of rehabilitation services will be critical in preventing another public health crisis after acute COVID-19 infection.

## Introduction

Coronavirus disease 2019 (COVID-19) is caused by severe acute respiratory coronavirus-2 (SARS Cov-2) [1]. The novel coronavirus is a single-stranded ribonucleic acid (RNA) virus, primarily affecting the respiratory system. Coronavirus pandemic has caused a major impact on health services, globally. The virus is transmitted from person to person via liquid droplets (sneeze, hand to mouth/eye contact, and contaminated surface) [2]. It has a wide range of presentation. The majority of affected individuals are asymptomatic. In patients with symptoms, it can present as mild common cold to severe life-threatening acute respiratory syndrome.

Initially, it was thought that the disease was acute in nature but now it is well known that it has chronic component too and may be associated with major disabilities. Now the terms like post-COVID-19 syndrome/sequelae or long COVID-19 are becoming common. In many cases, symptoms like fatigue and dyspnea are common even after three months of the onset of the disease. This can cause a significant impact on the activity of daily living. Apart from these, the involvement of the musculoskeletal system, gastrointestinal system, and nervous system makes the disease more complex and increases the patient’s overall morbidity. COVID-19 infection has the ability to grossly impact the psychological well-being of patients.

Initially, chronic sequelae of COVID-19 were not an expected outcome. But now its awareness is growing. Till now, there is little information on the extent of disabilities produced in COVID-19 survivors. This study was done to assess the extent of disability following COVID-19 infection using World Health Organization Disability Assessment Schedule 2.0 (WHODAS 2.0).

## Materials and methods

This cross-sectional study with convenient sampling was conducted in a tertiary care institute in Western India from November 2020 to May 2021. Enrollment of patients was done in the Out-Patient Department of Physical Medicine and Rehabilitation. Institutional ethical clearance was taken before the commencement of the study. Informed consent was taken from all patients.

All patients who recovered after COVID-19 infection [initial infection confirmed by real time polymerase chain reaction (RT-PCR) test] with age 18 years or more and at least three months’ duration post disease were recruited in the study. Patients who already had neurological and musculoskeletal disabilities (before COVID-19 infection) and were on medications for any psychiatric illness were also excluded from the study.

All patient’s data were collected by strictly following institutional COVID-19 prevention guidelines. All interviews were conducted in strict privacy and precaution was taken to avoid any emotional distress to participants. The demographic data were collected. Data of pre-existing comorbidity and symptom profile during COVID-19 infection were also collected. World Health Organization Disability Assessment Schedule 2.0 (WHODAS 2.0) was administered to all patients.

The WHODAS 2.0 scores were used to assess for health and disability. It covers six domains (total of 36 questions) of disability -- understanding and communicating (cognition), getting around, self-care, getting along with people, life activities, and participation in society. The total score ranges from 0 to 100%. Zero means no disability, while 100% means maximum disability. It also has a three-set questionnaire after the domain evaluation. All these domains assess various difficulties that the patient suffered within the last 30 days [3].

Single breath count (SBC) is a simple, non-invasive test to assess pulmonary function. In this patients were asked to do maximal inhalation and then start counting numbers from one in normal speaking tone and speed [4]. Total count is noted when patients try to inhale again.

The fatigue severity scale (FSS) was used to assess the severity of fatigue. FSS is a nine-item scale. Each item has a Likert rating scale that ranges from 1 to 7, where 1 is strongly disagree and 7 is strongly agree. Score greater than 4 indicates the presence of fatigue [5].

Statistical analysis

The data were entered into the Microsoft Excel database and were analyzed using IBM SPSS version 20 (IBM Corp., Armonk, NY). Descriptive statistics was used for continuous variables such as mean, standard deviation, and median. For categorical variables, it was represented as frequencies and proportions. Shapiro Wilk test was also used to see the normality of data. Non-parametric tests used as data were not normally distributed. Intergroup comparisons based on sex, diabetes status, hypertension status, hospitalization, and oxygen requirement were done using the Mann-Whitney test while body mass index (BMI) was compared using the Kruskal-Wallis test. For taking out the correlation between two variables, Spearman Correlation test was used. A p-value <0.05 was considered significant.

## Results

In total 97 COVID-19 survivors (CS) were recruited in the study. The mean duration of symptoms was 15.5 ± 3.64 weeks that ranged from 12 weeks to 33 weeks. The general baseline characteristics of the study population (continuous variables) are tabulated in Table 1. There was a male preponderance (63.9%) in the study population. The categorical variables are tabulated in Table 2.

**Table 1:** General baseline characteristics of study population. WHODAS 2.0, WHO Disability Assessment Schedule 2.0; SD, standard deviation

	N	Minimum	Maximum	Mean	SD
Age (years)	97	18.00	84.00	48.69	15.58
Weight (kg)	97	39.00	124.00	74.28	14.73
Height (cm)	97	134.60	188.00	165.42	12.11
Body mass index (kg/m^2^)	97	16.44	41.07	27.18	5.08
Duration (weeks)	97	12.00	33.00	15.50	3.64
Duration of admission in hospital (days)	97	0	30.00	5.08	6.97
Single-breath count	97	5.00	90.00	43.58	18.39
WHODAS 2.0%	97	0	61.11	11.62	16.43
Fatigue severity score	97	1.00	7.00	3.22	1.96
Understanding and communicating %	97	0	58.33	10.51	14.31
Getting around %	97	0	100.00	16.18	18.69
Self-care %	97	0	62.50	2.75	9.08
Getting along with people %	97	0	50.00	4.48	9.93
Life activities %	97	0	100.00	11.24	18.49
Participation in society %	97	0	90.62	13.85	16.78

**Table 2:** General characteristics of study population as frequencies.

Characteristic	Category	No. of subjects (%)
Sex	Male	62 (63.9%)
	Female	35 (36.1%)
Body mass index	Normal	38 (39.2%)
	Pre-obese	27 (27.8%)
	obese	32 (33.0%)
Diabetes mellitus	Diabetic	26 (26.8%)
Hypertension	Hypertensive	41 (42.3%)
Hospitalization	Hospitalized	47 (48.5%)
	Not hospitalized	50 (51.5%)
Oxygen requirement	Oxygen required	34 (35.1%)
Cardiac disease	-	8 (8.2%)
Pulmonary disease	-	5 (5.2%)
Alcohol	-	12 (12.4%)
Tobacco	-	7 (7.2%)
Smokers	-	4 (4.12%)

Symptomatology and course during COVID-19

Patients were asked about the symptoms/signs they had during active COVID-19 infection. Fever (68%) was the most common complaint followed by fatigue (67%) and cough (56.7%). Dyspnea (50.5%) and sore throat (41.2%) were other common complaints reported by patients. Many other symptoms were also reported by patients in the acute phase of COVID-19 infection, tabulated in Table 3.

**Table 3:** Symptoms during acute COVID-19 infection.

Symptoms during COVID-19	No. of patients (%)
Fever	66 (68%)
Fatigue	65 (67%)
Cough (total)	55 (56.7%)
Dry cough	41 (42.3%)
Wet cough	14 (14.4%)
Dyspnea	49 (50.5%)
Sore throat	40 (41.2%)
Myalgia	38 (39.2%)
Anorexia	31 (32%)
Rhinorrhea	24 (24.7%)
Headache	20 (20.6%)
Chest tightness	19 (19.6%)
Abdominal pain	16 (16.5%)
Anosmia	16 (16.5%)
Nausea	12 (12.4%)
Diarrhea	12 (12.4%)
Vomiting	8 (8.2%)
Confusion	2 (2.1%)
Hemoptysis	0

Around half of the patients (48.5%) were hospitalized during the initial COVID-19 infection and the rest (51.5%) were home quarantined. Some 35.1% of patients required oxygen. Ventilator support was utilized in 8.51% of patients among hospitalized patients. The mean duration of hospital stay for the admitted patient was 10.49 ± 6.61 days (median 9 days) which ranged from 2 to 30 days. Among the admitted patients, 14.89% received remdesivir.

Symptomology in post-COVID-19 period (after three months of infection)

Fatigue was the most common complaint (61.9%) reported by COVID-19 survivors after three months. Among them, 73.33% of patients had persistent fatigue while 26.67% of COVID-19 survivors developed fatigue later.

Dyspnea was the second most common problem reported by COVID-19 survivors. It was present in 56.7% of patients. Patients had specific musculoskeletal problems (37.1%) that included low back pain, upper back pain, cervical pain, joint pains (ankle, knee, shoulder, elbow, and small joints of hand), and muscular tenderness. Generalized body ache (7.2%) was also found to be impacting the patient’s life. Other reported complaints were difficulty in sleeping, generalized body ache, abdominal pain, headache, persistent cough, chest pain, vertigo, nausea, and epistaxis (Table 4).

**Table 4:** Symptoms after three months of COVID-19 infection.

Post-COVID-19 symptoms	No. of patients (%)
Fatigue	60 (61.9%)
Dyspnea	56 (56.7%)
Musculoskeletal complaints	36 (37.1%)
Difficulty in sleeping	11 (11.3%)
Generalized Body Ache	7 (7.2%)
Abdominal Pain	5 (5.2%)
Headache	4 (4.1%)
Persistent cough	1 (1%)
New-onset cough	3 (3.1%)
Chest pain	3 (3.1%)
Vertigo	1 (1%)
Nausea	1 (1%)
Epistaxis	1 (1%)

Among initial asymptomatic patients, dyspnea and fatigue were the most commonly reported post-COVID-19 symptoms. Seven patients had dyspnea and six patients had fatigue (both were present in five patients). Abdominal pain, sleeping difficulties, wet cough, and joint pain were other symptoms reported by these patients.

Disability assessment using WHODAS 2.0

Intergroup comparison between median values of WHODAS 2.0 based on sex (male/female), BMI (normal/pre-obese/obese), diabetes status (diabetic/non-diabetic), hypertension status (hypertensive/non-hypertensive), hospitalized (hospitalized/non-hospitalized), and oxygen requirement during initial COVID-19 presentation (oxygen required/oxygen not required) were done (Table 5).

**Table 5:** Comparison of WHODAS 2.0 score among various categories. WHODAS 2.0, WHO Disability Assessment Schedule 2.0

Characteristic	Groups	N (%)	Median	WHODAS 2.0		
				Mean rank	Statistic	p-value
Sex	Male	62 (63.9%)	3.12	43.11	*Z*=0-2.74	0.006
	Female	35 (36.1%)	12.50	59.43		
Body mass index	Normal	38 (39.2%)	7.29	48.64	Chi-Square: 1.57 df = 2	0.456
	Pre-obese	27 (27.8%)	2.77	44.26		
	Obese	32 (33.0%)	9.02	53.42		
Diabetes	Diabetic	26 (26.8%)	11.10	54.33	Z= -1.13	0.258
	Non-diabetic	71 (73.2%)	6.25	47.05		
Hypertension	Hypertensive	41 (42.3%)	6.25	47.48	Z= -0.46	0.646
	Not hypertensive	56 (57.7%)	7.64	50.12		
Hospitalization	Hospitalized	47 (48.5%)	9.02	50.82	Z= -0.62	0.536
	Not hospitalized	50 (51.5%)	5.90	47.29		
Oxygen requirement	Oxygen required	34 (35.1%)	8.68	49.28	Z= -0.07	0.943
	Oxygen not required	63 (64.9%)	6.25	48.85		

There was a significant difference between mean values of WHODAS 2.0 between males and females. Females had a higher disability in comparison to males. Among domains, significant difference in means was found in getting around (mean rank male/female 41.88/61.61, *Z*=-3.376, p-value=0.001), self-care (mean rank male/female 45.85/54.59, *Z*=-2.220, p-value=0.026), life activities (mean rank male/female 44.36/57.21, *Z*=-2.227, p-value=0.026), and participation in society (mean rank male/female 43.69/58.41, *Z*=-2.525, p-value=0.012). There was a significant disability in understanding and communication in non-hypertension subgroups (mean rank hypertensive/non-hypertensive 42.26/53.94, *Z*=-2.106, p-value=0.035). There was no significant difference in medians of disability between other groups of WHODAS 2.0 and its domains (Table 6).

**Table 6:** Comparison of WHODAS 2.0 domains. WHODAS 2.0, WHO Disability Assessment Schedule 2.0 D1: understanding and communicating (cognition); D2: getting around; D3: self-care; D4: getting along with people; D5: life activities; D6: participation in society

Characteristics	Groups	Median	p-value					
			D1	D2	D3	D4	D5	D6
Sex	Male	3.12	0.554	0.001	0.026	0.606	0.026	0.012
	Female	12.50						
Body mass index	Normal	7.29	0.594	0.072	0.832	0.486	0.254	0.688
	Pre-obese	2.77						
	Obese	9.02						
Diabetes	Diabetic	11.10	0.422	0.512	0.658	0.800	0.568	0.053
	Non-diabetic	6.25						
Hypertension	Hypertensive	6.25	0.035	0.744	0.982	0.284	0.477	0.763
	Not hypertensive	7.64						
Hospitalization	Hospitalized	9.02	0.532	0.678	0.145	0.508	0.849	0.195
	Not hospitalized	5.90						
Oxygen requirement	Oxygen required	8.68	0.822	0.678	0.954	0.693	0.882	0.555
	Oxygen not required	6.25						

The WHODAS 2.0 also has three questions at the end of the assessment in which the patient has to notify the number of days in the last 30 days he had problems as asked in questions. The questions were: overall, in the past 30 days, how many days were these difficulties present? (H1), In the past 30 days, for how many days were you totally unable to carry out your usual activities or work because of any health condition? (H2) and in the past 30 days, not counting the days that you were totally unable, for how many days did you cut back or reduce your usual activities or work because of any health condition? (H3). Zero days difficulties in all H1, H2, and H3 were reported in 37.11% patients (all scores were zero) [3]. Separately, 39.17%, 79.38%, and 67.01% of patients reported zero days difficulty in H1, H2, and H3 respectively. Mean and standard deviation (with median) scores of H1, H2, and H3 were 13.22 ± 13.09 days (10), 3.01 ± 7.51 days (0), and 5.12 ± 9.32 days (0) respectively.

Comparisons of WHODAS 2.0, SBC, and FSS in patients with and without fatigue, dyspnea, and musculoskeletal complaints are tabulated in Table 7. Correlations of WHODAS 2.0 with fatigue severity score and single breath count are tabulated in Table 8.

**Table 7:** Comparison of WHODAS 2.0, SBC, and FSS in patients with and without fatigue, dyspnea, and MSK complaints. N, no. of patients; SBC, single breath count; FSS, fatigue severity scale; WHODAS 2.0, WHO Disability Assessment Schedule 2.0; MSK, musculoskeletal

Symptoms	Group	N	SBC statistic	FSS statistic	WHODAS 2.0 statistic
Fatigue	Present	60	*Z*=0.3.423 p=0.001	*Z*= -6.473 p<0.001	*Z*= -4.911 p<0.001
	Absent	37			
Dyspnea	Present	56	*Z*=0.2.36 p=0.018	*Z*= -4.918 p<0.001	*Z*= -4.287 p<0.001
	Absent	41			
MSK complaints	Present	36	*Z*=-1.2 p= 0.230	*Z*= -1.03 p= 0.303	*Z*= -0.5 p= 0.611
	Absent	61			

**Table 8:** Correlation of WHODAS 2.0, fatigue severity score, and SBC. WHODAS 2.0, WHO Disability Assessment Schedule 2.0; SBC, single breath count

		Single breath count	WHODAS 2.0%	Fatigue severity score
Single breath count	Correlation coefficient	1.000	-0.358	-0.465
	p-value	-	< 0.0001	< 0.0001
WHODAS 2.0%	Correlation coefficient	-0.358	1.000	0.736
	Sig. (two-tailed)	0.000	-	0.000
Fatigue severity score	Correlation coefficient	-0.465	0.736	1.000
	p-value	<0.0001	< 0.0001	-

## Discussion

Post-COVID-19 sequelae or long COVID-19 is a complex, multisystem illness. Long COVID-19 is delayed recovery from an episode of COVID-19 and is characterized by the lasting effect of infection [6]. This persistence of symptoms and appearances of new symptoms in COVID-19 survivors leads to significant disability.

A combination of fatigue and dyspnea becomes very problematic in managing activities of daily living (ADL) for the patient. Fatigue after COVID-19 is found not to be associated with the initial severity of disease and CT thorax abnormalities [7]. The exact molecular mechanisms of the development of fatigue are not yet known. Dyspnea may be attributable to fibrotic changes in the lung after COVID-19 [8].

Fatigue is one of the most commonly reported and most poorly understood symptoms even after a few months of getting COVID-19 [9]. Many studies reported fatigue in 24%-63% of the study population even after three to six months [7-8, 10-12]. This fatigue may be persistent or develops after a few months of COVID-19. In our study, fatigue is reported by 61.9% of the patients. Among them, 26.67% of patients developed fatigue a few months after recovering from COVID-19. Persistent fatigue affected 73.33% of patients. The majority of patients continued to suffer from fatigue that developed during active COVID-19. Fatigue may be due to general deconditioning, associated weakness, and psychological stress.

Both dyspnea and persistent cough are indicators of impaired lung function [13]. Post-COVID-19 dyspnea is another common symptom reported in our study. More than half of the patients complained of dyspnea. Among them, 55.36% of COVID-19 survivors had persistent dyspnea and 44.66% developed dyspnea after a few months. Persistent cough was reported by only 1% and new-onset cough by 3.1% of COVID-19 survivors. 

Ali et al. in 2011 reported a positive correlation of SBC with forced vital capacity (FVC) and FVC at 1 s. SBC can reflect both restrictive and obstructive pulmonary disease [4]. In our study, there was a significant difference between medians of WHODAS 2.0, SBC, and FSS in patients with and without fatigue or dyspnea. COVID-19 survivors who had fatigue or dyspnea had more disability than patients without them. There was a strong positive correlation between WHODAS 2.0 and fatigue severity score and a weak negative correlation with SBC. A moderate negative correlation was also observed between FSS and SBC. These correlations suggest that patients with high FSS or low SBC have more disabilities.

In the Indian scenario, most of the household work is dependent on females of the house. Lockdown increased this burden on females. Closure of schools, caregiving to family members, economic burden due to loss of job or unpaid work, and increased burden on females. During the outbreak, their needs were largely unmet as in most households they lacked power in decision making [14]. In our study, females had a significantly higher post-COVID-19 disability as compared to males. Among WHODAS 2.0 domains, females had a significantly higher disability in getting around, self-care, life activities, and participation in society.

New onset musculoskeletal complaints are another area of concern in COVID-19 survivors in our study. Low back pain, upper back pain, cervical pain, joint stiffness, and pain were major issues. Headache, abdominal pain, and chest pain may affect COVID-19 survivors. In our study, there was no significant difference between medians of WHODAS 2.0, SBC, and FSS in patients with or without musculoskeletal complaints.

Psychological distress due to persistent symptoms or new symptoms after COVID-19 may lead to sleep disturbances. Change in the sleep-wake cycle may also be due to quarantine, lockdown, decreased physical activity, and excessive social media exposure [15]. In our study, 11.3% of COVID-19 survivors had difficulty in sleeping.

Hypertension, diabetes, and high body mass index are risk factors for the high severity of acute COVID-19 [16-18]. All these patients along with patients who were hospitalized or required oxygen were expected to have more disability later. But in our study, we did not find any significant difference in overall post-COVID-19 disability based on BMI, diabetes, hospitalization, oxygen requirement, and hypertension. Although in the cognition domain of WHODAS 2.0, the non-hypertensive group had more disability. More studies are required to confirm this result.

Recognition of post-COVID-19 sequelae and availability of rehabilitation services will be critical in preventing another public health crisis after acute COVID-19. To counteract post-COVID-19 disability, cardiac, pulmonary, and musculoskeletal rehabilitation of COVID-19 survivors become the topmost priority as the burden of disease may increase with time. All COVID-19 survivors should be screened for any COVID-19-related disability. A rehabilitation program must be made keeping in mind the exclusion criteria of physical rehabilitation as in many survivors of severe COVID-19 critical illness with severe pulmonary and cardiac damage, physical rehabilitation is not suitable [19]. Individualized rehabilitation programs which should be tailored by physiatrist and patient managed by multidisciplinary team may reduce the burden of post-COVID-19 disability though robust research is required for its effectiveness.

There are some limitations to our study. The study was cross-sectional in nature, time-bound, and done in a single center. The study population was limited to the patients coming for treatment to a tertiary care center (also a major COVID-19 hospital) in a state of Western India.

## Conclusions

Fatigue followed by dyspnea was the most common symptom reported in post-COVID-19 survivors. COVID-19 survivors with fatigue or dyspnea had a more significant disability as compared to other patients. Females had a more significant difference in the disability when compared to males. We did not find any significant disability in COVID-19 survivors after three months of disease based on BMI, hospitalization, diabetes, and oxygen requirements. We recommend that all COVID-19 survivors should be educated early about these symptoms and disabilities they can produce and a regular follow-up should be done based on telemedicine or the outpatient department model.
